# Epidemiology of suicide: from population to single cases

**DOI:** 10.1017/S2045796019000647

**Published:** 2019-11-11

**Authors:** Maurizio Pompili

**Affiliations:** Department of Neurosciences, Mental Health and Sensory Organs, Suicide Prevention Center, Sant'Andrea Hospital, Sapienza University of Rome, Rome, Italy

**Keywords:** Education psychiatric, epidemiology, mental health, suicide

The new millennium was inaugurated with pessimistic figures on the epidemiology of suicide for years to come. Based on the collections of mortality rates from the World Health Organization (WHO), it was proposed that the one million deaths from suicide registered annually worldwide could rise to 1.56 million deaths annually by the year 2020 (Bertolote and Fleischmann, 2002). Such reports also showed the dramatic changes among year-groups, pointing to an increase in suicide rates among youths and young adults compared with people over the age of 45 years, who were the majority in the 1950s. As the years passed, such figures had to be corrected and there was a resizing of the original projections, with a reduction of suicide deaths worldwide (Bertolote and De Leo, 2012). In 2014, WHO (World Health Organization, 2014) released a comprehensive analysis of suicides in the world and indicated that 880 000 suicide deaths were registered annually. Of note, this report, which was awaited for some years, proposed basic but fundamental elements for suicide prevention, such as the difference between myths and fact about suicide. Such notions are well-known to suicidologists but are often neglected both in healthcare and among laypeople. For instance, a myth is ‘Only people with mental disorders are suicidal’, whereas the fact is ‘Suicidal behaviour indicates deep unhappiness but not necessarily mental disorder. Many laypeople living with mental disorders are not affected by suicidal behaviour, and not all people who take their own lives have a mental disorder’; another myth is ‘Most suicides happen suddenly without warning’, whereas the fact is ‘The majority of suicides have been preceded by warning signs, whether verbal or behavioural. Of course, there are some suicides that occur without warning. But it is important to understand what the warning signs are and look out for them.’ Another major contribution to this report is the identification of key risk factors for suicide (factors related to society, community, relationships and the individual), which are aligned with the relevant interventions. Of note is the fact that mental disorders appear among many other circumstances and are also listed as individual risk factors. In the international literature, variables that may impact on individuals with different emphasis are generally left out of any discussion. New understanding of suicidal behaviour is now leading research beyond the statement that 90% of suicides were due to mental disorders. Incidentally, the phenomenon of suicide is much more complicated than the attribution of risk to a given disorder. It is more likely to be the result of a drama occurring in different ways for each unique individual. Therefore modern psychiatry is now changing such a paradigm and a different approach must be considered when it comes to suicide prevention, in line with recent epidemiological data. For instance, The Italian National Institute of Statistics (ISTAT, 2017) conducted a study on suicide in the Italian population for the 3 years 2011–2013, evaluating the presence of any physical or mental disorders. In the period considered there were 12,877 suicides (of which 10,065 were men), and about one in five suicides had significantly associated morbidities. In particular, in 737 suicides, the presence of relevant physical diseases was documented, of which 288 had a comorbid mental disorder. In 1664 cases, the study reported the presence of mental disorders without comorbidity from relevant organic diseases. However, in more than 80% of the cases, there were neither mental disorders nor relevant physical illnesses. Similarly, the Centers for Disease Control and Prevention in the USA (CDC) found a 30% increase in suicides across all age groups up to age 75 years between 1999 and 2016, indicating that about 54% of cases in 2015 were not associated with any mental disorder (Stone *et al*., 2018). Some scholars suggest that people with no psychiatric diagnosis did not have any chance to be assessed by mental health services. Nevertheless, the CDC has launched an information campaign to shed light on suicide, pointing to contributing factors other than mental illness (https://www.cdc.gov/vitalsigns/suicide/index.html). They emphasised that any single factor rarely causes suicide and that the role of relationship problems, substance misuse, recent crisis and job, financial or legal stress should be taken into consideration. Such conclusions had been proposed much earlier by scholars who estimated that effective treatment of the psychiatric disorders commonly associated with suicide risk would have reduced suicide rates by only a small degree (Bertolote *et al*., 2003, 2004). These authors suggested broader evaluation of factors associated with such a risk and the implementation of several suicide prevention strategies.

Of interest for such discussion is what the fifth edition of the *Diagnostic and Statistical Manual of Mental Disorders* (DSM-5) proposed in its introduction (American Psychiatric Association, 2013). This manual warned that: ‘*Diagnosis of a mental disorder should have clinical utility*’ but ‘*the diagnosis of a mental disorder is not equivalent to a need for treatment. Need for treatment is a complex clinical decision that takes into consideration symptom severity, symptom salience (e.g. the presence of suicidal ideation), the patient*'*s distress (mental pain)*’ and ‘*Clinicians may thus encounter individuals whose symptoms do not meet full criteria for a mental disorder but who demonstrate a clear need for treatment or care. The fact that some individuals do not show all symptoms indicative of a diagnosis should not be used to justify limiting their access to appropriate care*’ (p. 20).

Being both a psychiatrist and a suicidologist, I usually meet many patients who desperately need a reduction of their mental pain, regardless of the diagnosis. Shneidman (1993) believed that, in suicide, ‘death’ is not the keyword. The keyword is ‘mental pain’ and if the pain were relieved then the individual would be willing to continue to live. The understanding of the suicidal mind requires knowledge of the perturbed state of the individual in crisis because this provides the motivation for the individual to contemplate suicide. The subject at risk feels hopeless and the mental pain feels unique, and he/she reaches this conclusion after experiencing not being able to communicate this suffering to the people assigned to help. The desire to die happens in each person with substantially unique motivations and thoughts, which makes him/her different from all other people at risk of suicide. Considering suicide risk to be merely a symptom impairs the opportunity to fully investigate and understand suicide (Pompili, 2019). Attempts to explain, predict and control suicide require an understanding of what suicidal thoughts and feelings mean to those who live it. To distinguish suicidal content from psychiatric diagnosis, it is necessary to realize that the elements that support the desire to die constitute a process in its own right, with a logic typical of the mind that suffers and that tries to devise a solution to reduce or resolve this suffering. Among the myths often cited to describe idiosyncrasies in the phenomenon of suicidal behaviour, classical suicidology and current opinion state that people who talk about killing themselves rarely die by suicide, whereas in fact most people who die by suicide have given some verbal clue or warning of their intentions (Pompili *et al*., 2016).

Two editorials in this issue focus on hot issues of suicide prevention. Professor Baldessarini (Baldessarini 2019) analyzes recent epidemiological data and points to proper assessment and treatment of the suicidal individual; he reports recent findings regarding suicide risk in patients with mood disorders. Furthermore, he highlights features associated with suicide risk among patients suffering from bipolar disorders, which can help clinicians to recognise individuals at risk. He also points to the issue of suicide risk in the post-discharge period, as well as results indicating that suicide risk is concentrated in the first period after the onset of bipolar disorders. In this regard, pharmacological treatments of suicide risk remain limited, although consolidated evidence is available for some drugs.

In the second editorial, Professor Pirkis (Pirkis *et al*., 2019) and colleagues overview some of the pitfalls and biases of conducting studies on suicide. They highlight the power and limitations of psychological autopsy studies, a method of investigation that involves and in some way supports people who have lost a dear one by suicide. They present the pros and cons of the case–control studies, which seem to provide a solid guarantee for collecting such data. Furthermore, they point to register-based studies as a comprehensive and systematic collection of important data but these can leave out variables that are important for understanding suicidal behaviour.

In conclusion, based on my thoughts and those reported in the editorials, there is the need to reconceptualise the study of suicide with innovative understanding by taking account of clinical, epidemiological and phenomenological aspects of suicide. Future studies should consider both epidemiology and statistics, and should also reach clinicians with proper education for dealing with a suicidal individual. The challenge of the present time is to inform and educate on suicide prevention and thus create a new culture in mental health facilities, which can become safe havens for those thinking about suicide.
